# Potentials of Cellular Reprogramming as a Novel Strategy for Neuroregeneration

**DOI:** 10.3389/fncel.2018.00460

**Published:** 2018-11-30

**Authors:** Lyujie Fang, Layal El Wazan, Christine Tan, Tu Nguyen, Sandy S. C. Hung, Alex W. Hewitt, Raymond C. B. Wong

**Affiliations:** ^1^Centre for Eye Research Australia, East Melbourne, VIC, Australia; ^2^Ophthalmology, Department of Surgery, The University of Melbourne, Melbourne, VIC, Australia; ^3^Department of Ophthalmology, Jinan University, Guangzhou, China; ^4^Menzies Institute for Medical Research, University of Tasmania, Hobart, TAS, Australia; ^5^Shenzhen Eye Hospital, Shenzhen, China

**Keywords:** cell reprogramming, retina, neuroregeneration, direct reprogramming, *in vivo* reprogramming, regenerative medicine, gene therapeutics

## Abstract

Cellular reprogramming technology holds great potential for tissue repair and regeneration to replace cells that are lost due to diseases or injuries. In addition to the landmark discovery of induced pluripotent stem cells, advances in cellular reprogramming allow the direct lineage conversion of one somatic cell type to another using defined transcription factors. This direct reprogramming technology represents a rapid way to generate target cells in the laboratory, which can be used for transplantation and studies of biology and diseases. More importantly, recent work has demonstrated the exciting application of direct reprogramming to stimulate regeneration *in vivo*, providing an alternative approach to transplantation of donor cells. Here, we provide an overview of the underlying concept of using cellular reprogramming to convert cell fates and discuss the current advances in cellular reprogramming both *in vitro* and *in vivo,* with particular focuses on the neural and retinal systems. We also discuss the potential of *in vivo* reprogramming in regenerative medicine, the challenges and potential solutions to translate this technology to the clinic.

## Background

During development, cellular identity, and differentiation potential are largely determined by the lineage history of the specific cell. Generally, the cell identity or the differentiated state of an adult cell is remarkably stable, and only stem cells with multipotent/pluripotent potential possess the ability to turn into another cell type(s). By transferring the somatic cell nucleus into an enucleated oocyte in Xenopus, the Nobel Prize-winning work of Gurdon (1962) first demonstrated that mature cells can be reprogrammed back to an embryonic state and became pluripotent. Similar nuclear transfer was subsequently demonstrated in mammals, including Dolly the cloned sheep. These pioneering studies provided evidence that reprogramming factors in the oocyte cytoplasm can overwrite the cellular identity encoded in the nucleus of a fully differentiated cell (Campbell et al., 1996). However, the precise factors that enables cell fate conversion in mature cells remain largely elusive. The seminal work of Shinya Yamanaka’s group identified the precise signals required for cellular reprogramming, and showed that a cocktail of four transcription factors (*Oct4*, *Sox2*, *c-Myc,* and *Klf4)* are sufficient to reprogram skin fibroblasts into induced pluripotent stem (iPS) cells that resemble a primitive embryonic state (Takahashi and Yamanaka, 2006). Since then, there has been intensive research on the identification of the precise transcription factors to alter and reprogram cell fates.

Beyond iPS cell reprogramming, direct conversion of one somatic cell type to an unrelated cell type can occur without passing through an intermediate multipotent state. This direct reprogramming approach, also known as ‘transdifferentiation,’ was first demonstrated with the conversion of fibroblasts into myoblasts by overexpression of a single transcription factor *MyoD*. This highlighted the potential power of controlling cell fates using genetic factors (Davis et al., 1987). Another early example of direct reprogramming was conducted by Kulessa et al. (1995) who successfully reprogrammed myoblasts into eosinophils and thromboblasts by forceful overexpressing of *GATA1*.

**FIGURE 1:**
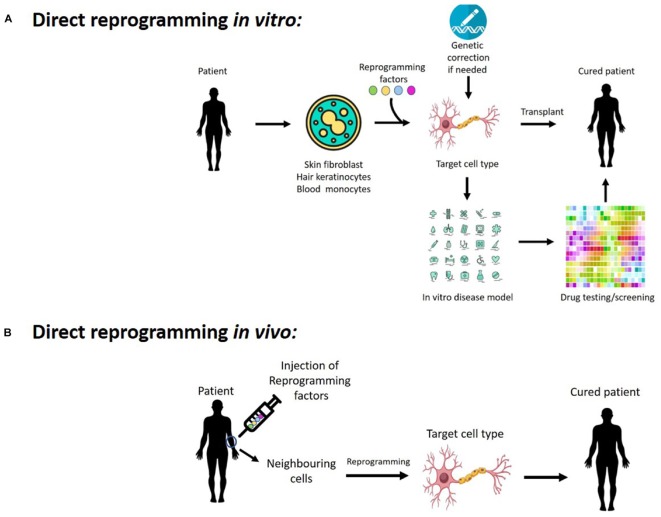
Potentials of cellular reprogramming **(A)** *in vitro* and **(B)** *in vivo* for regenerative medicine, disease modeling, as well as drug discovery and testing gene therapy.

In recent years, direct reprogramming has attracted enormous interest, yielding promising results in the conversion across multiple lineages to generate neurons, oligodendrocytes, cardiomyocytes, muscle cells, blood progenitors, and hepatocytes (Ladewig et al., 2013). Notably, this direct reprogramming process is potentially much faster than generating iPS cells and subsequent differentiation into the target cell types, which could take months. Thus, it represents a faster and more cost-effective method to generate cells *in vitro*. The concept of taking patient’s cells and turning them into a different cell type is very attractive for tissue engineering and regenerative medicine, as it eliminates the risk of immune-rejection of the reprogrammed cells following transplantation. This direct reprogramming approach also has the potential to generate disease models using the patient’s cells, allowing the study of disease mechanism as well as providing an *in vitro* platform for drug discovery, toxicology study and testing of gene therapy (Figure 1A). Moreover, the application of direct reprogramming *in vivo* would allow us to reprogram endogenous cells within the body to become new cell types, providing a novel strategy to regenerate cells that are lost in diseases or injuries (Figure 1B).

In this review we will discuss both the *in vitro* and *in vivo* application of direct reprogramming, its clinical implementation in regenerative medicine, the future directions and the current challenges to translate this technology into clinical practice.

## Direct Reprogramming to Generate Neurons *In Vitro*

The underlying concept of cellular reprogramming is that the transcriptome plays an important role in defining cellular identity, hence alteration of the transcriptome to a profile specific to the target cell type would allow us to control and convert cell fate. Many reprogramming studies utilized a ‘defined transcription factors’ approach, where a combination of transcription factors, usually master regulators during development, were used to alter the transcriptome and effectively reprogram cell fates. Here we will discuss recent reprogramming studies in conversion of cell lineages, with a focus on transcription factor-based reprogramming studies. However, it should be noted that other reprogramming approaches have also been described, such as the use of microRNA and small molecules for cellular reprogramming which were reviewed in details recently by others (Janowska et al., 2018; Li et al., 2018; Lu and Yoo, 2018).

In a landmark study, Vierbuchen et al. (2010) showed that *Ascl1*, a proneural master regulator in neuron specification, can be used in combination with two other factors (*Brn2, Mytl1)*, to reprogram mouse fibroblasts into induced Neurons (iN). These iN expressed neuronal markers and have the ability to form functional synapses (Vierbuchen et al., 2010). Subsequent studies demonstrated the generation of iN from human embryonic fibroblast, using *ASCL1, BRN2, MYTL1* with the addition of *NEUROD1* (Pang et al., 2011), which is a helix-loop-helix transcription factor that plays an important role in neuronal development. Notably, the derived human iN are capable of synapse formation and possess a functional electrophysiological profile indicative of immature neurons. Further study demonstrated that *Ascl1* solely is sufficient to reprogram fibroblasts to excitatory iN (Chanda et al., 2014). These results established *Ascl1* as a powerful reprogramming factor for iN generation. Although *Brn2* and *Mytl1* are not required for iN reprogramming, the two factors play a role in enhancing early maturation of iN reprogramming.

It should be noted that the derived iN are often a mixed population of neuron subtypes, often a mix of GABAergic or glutamatergic neurons. Other transcription factors have been identified for direct reprogramming into neuronal subtypes. For instance, both human and mouse fibroblasts can be reprogrammed into dopaminergic neurons with functional electrophysiology using *Ascl1, Nurr1,* and *Lmx1a* (Caiazzo et al., 2011). Importantly, this reprogramming process has been used to generate dopaminergic neurons from a Parkinson’s disease patient, which demonstrated the potential to use direct reprogramming for disease modeling (Figure 1A). Other studies have identified additional reprogramming factors for dopaminergic neuron reprogramming, including *Foxa2, Brn2* (Pfisterer et al., 2011) and *Pitx3* (Kim et al., 2011). Moreover, fibroblasts can be reprogrammed into dopaminergic neurons using a cocktail of five transcription factors, *ASCL1, NGN2, SOX2, NURR1* and *PITX3*, with a reported 40% efficiency in generation of dopaminergic neuron-like cells positive for dopa decarboxylase expression. The reprogrammed neurons exhibited an electrophysiological profile that is dependent of the D2 autoreceptor, thus providing evidence that the reprogrammed neurons are functionally similar to dopaminergic neurons (Liu et al., 2012). In another example, motor neurons can be generated using a similar direct reprogramming strategy. Son et al. (2011) demonstrated that a complex mix of *Ascl1, Brn2, Mytl1, Ngn2, Lhx3, Isl1,* and *Hb9 or Neurod1* allow the reprogramming of fibroblasts into motor neurons. Additional work showed the role of *Sox11* in facilitating this reprogramming process (Liu et al., 2013). Similarly, the feasibility of generating sensory neurons has also been demonstrated using direct reprogramming. *Brn3a, Ngn1,* or *Ngn2* allowed the reprogramming of fibroblasts into peripheral sensory neurons that include a subset that respond similarly to itch- and pain-sensing neurons (Blanchard et al., 2015). On the other hand, *Ascl1, Brn2, Mytl1, Ngn1, Isl2,* and *Klf7* have been utilized for direct reprogramming to generate noxious stimulus-detecting neurons with a characteristic inflammatory peripheral sensitization response (Wainger et al., 2015).

Also, emerging evidences have supported feasibility of using miRNAs for cellular reprogramming. Similar to transcription factors, miRNAs are also able to target large networks of genes and epigenetic regulators of the chromatin (Rajman and Schratt, 2017). In particular, overexpression of the neural-enriched miRNA, miR-9/9^∗^ and miR-124, allowed the direct reprogramming of human fibroblasts into functional neurons (Yoo et al., 2011). This reprogramming process can be further enhanced by the addition of *NEUROD2,* *ASCL1* and *MYT1L,* which supported an important role of the miR-9/9^∗^-124 signaling in neuronal reprogramming. Furthermore, miRNA-mediated reprogramming to generate specific neuronal subtypes is also possible. A follow-up study by the same group showed that this miRNA-mediated reprogramming process can be directed to generate striatal medium spiny neurons, by addition of four other transcription factors: *BCL11B, DLX1, DLX2,* and *MYT1L* (Victor et al., 2014). On the other hand, co-expression of miR-9/9^∗^-124 with two other transcription factors, *ISL1* and *LHX3*, promote reprogramming of human fibroblasts to motor neurons (Abernathy et al., 2017). Similarly, miR124 can also be used with other transcription factors (*ASCL1, NURR1, LMX1A*) for neuronal reprogramming to generate human dopaminergic neurons (Jiang et al., 2015). Recent studies have provided insights into the mechanism by which miR-9/9^∗^-124 promote neuronal reprogramming. There are evidences to support that miR09/9^∗^-124 promote neuronal reprogramming by reducing activity of the transcriptional repressor REST, which in turn reconfigure chromatin accessibility to allow expression of neuronal genes (Abernathy et al., 2017; Drouin-Ouellet et al., 2017; Lee et al., 2018). Collectively, these studies support an important role of miRNA in the conversion of cell fate to neurons.

Furthermore, small molecules have been identified to complement transcription factor-based direct reprogramming. This includes Forskolin, an activator of adenylate cyclase resulting in an increase in c-AMP, as well as Dorsomorphin, an inhibitor of BMP signaling. The combination of these two small molecules has been demonstrated to enhance neuronal reprogramming using *NGN2* or *SOX11* (Liu et al., 2013). In addition, inhibition of SMAD signaling (SB431542 and noggin) and GSK3B signaling (CHIR99021) have also been shown to promote neuronal reprogramming (Ladewig et al., 2012). An important milestone was achieved in 2015, when HongKui Deng’s group developed an all chemical approach to convert mouse fibroblasts into induced neurons that doesn’t require genetic manipulation (Li et al., 2015). The authors demonstrated that the use of five small molecules (ISX9, SB431542, Forskolin, CHIR99021, I-BET151) was sufficient to rewrite the fibroblast-specific transcriptome, activate neuronal-related genes and effectively convert fibroblast into neurons with up to 90% efficiency in 16 days. Subsequent work by Hu et al. (2015) extended this all-chemical approach for neuronal reprogramming of human fibroblasts, using a cocktail of seven small molecules: valproic acid, CHIR99021, REPSOX, Forskolin, Sp600125, GO6983, and Y27632. This reprogramming method allowed derivation of human iN containing a mix of GABAergic, cholinergic and dopaminergic neurons, and provided an alternative approach for neuronal reprogramming without the aid of transcription factors.

As direct reprogramming allows the conversion of cells from distinct lineages, it is postulated that this reprogramming process would potentially be more efficient to convert cell fates across lineages that are closely related and more similar in nature, compared to lineages that are distant and markedly different. For instance, iPS cell generation using keratinocytes exhibits a much higher efficiency compared to using fibroblasts (Aasen et al., 2008; Piao et al., 2014). One reason for the increased efficiency can be attributed to the nature of fibroblasts, which go through a mesenchymal-to-epithelial transition in the process of iPS cell reprogramming (Li et al., 2010; Takahashi et al., 2014), whereas keratinocytes of ectodermal origin may not require that transition. Another cellular reprogramming example across related lineages is the conversion of glia to neurons. Early work by Heins et al. (2002) showed that overexpression of *Pax6*, a master regulator of neural specification, can induce neurogenesis in glial cells. Further work by Heinrich and colleagues identified *Ngn2* as a reprogramming factor to convert astroglia into glutamatergic neurons, while expression of *Dlx2* induced GABAergic neurons with mature dendrites. The reprogrammed cells expressed pan-neuronal markers (βIII tubulin, MAP2) while the glial marker GFAP was downregulated. Importantly, the reprogrammed neurons were capable of establishing synapses and possessed functional electrophysiology (Heinrich et al., 2010). Similarly, others have highlighted the feasibility of using small molecules to reprogram human fetal astrocytes into neurons (Zhang et al., 2015; Gao et al., 2017).

## Application of Direct Reprogramming to Convert Cell Lineages *In Vivo*

The prospect of using direct reprogramming to convert endogenous cells *in vivo* into the target cells that are lost in disease or injury provides an exciting approach for regenerative medicine (Figure 1B). This is an emerging area of research that is attracting enormous interest for its therapeutic potential (Srivastava and DeWitt, 2016; Wong and Nguyen, 2017). Notably, this approach bypasses many of the major obstacles posed by transplantation-based regenerative strategies, such as issues with immune rejection and the high cost involved in production of clinical grade cells for transplantation. Instead, *in vivo* reprogramming can be promoted by viral delivery of reprogramming factors, such as an injection of adeno-associated viruses (AAVs) to the patient. This strategy is substantially cheaper to produce, easier to store and often an easier clinical procedure compared to cell transplantation.

Early pioneering work has demonstrated the feasibility of *in vivo* reprogramming to convert cell fates in the pancreas and cardiac systems. A pioneering study by Zhou et al. (2008) showed the reprogramming of pancreatic exocrine cells into β-cells in diabetic adult mice by using a combination of three transcription factors (*Ngn3*, *Pdx1,* and *Mafa*). Remarkably, the induced β-cells improve insulin production in the diabetic mice, demonstrating a great potential for using direct reprogramming as a regenerative strategy to treat diabetes (Zhou et al., 2008). A subsequent study has identified the use of *Ngn3* and *Mafa* for *in vivo* reprogramming to convert pancreatic acinar cells into δ- or α-like endocrine cells (Li et al., 2014) and advanced the discovery of transcription factors that allow direct reprogramming to generate a panel of pancreatic cell types *in vivo*.

In the cardiac system, Qian et al. (2012) demonstrated that cardiac fibroblasts can be reprogrammed into cardiomyocytes *in vivo* by using transcription factors *Gata4*, *Mef2c,* and *Tbx5*. The induced cardiomyocytes possess similar characteristics of binucleation, sarcomere assembly, electrical coupling, and gene expression profile typical of cardiomyocytes. Using a mouse myocardial infarction model, the authors injected a retrovirus carrying GMT into the infarct site and found that the infarct area was diminished. Notably, about 35% of the cardiomyocytes in the marginal area of the infarct were reprogrammed from cardiac fibroblasts. The induced cardiomyocytes can express the functional characteristics of adult ventricular muscles; about half of them have an organized sarcomere structure that improves cardiac function 2–3 months after myocardial infarction. Concurrently, Song et al. (2012) showed the use of a similar cocktail (*Gata4, Mef2c, Hand2*, *Tbx5*) to successfully reprogram non-cardiomyocytes into induced cardiomyocytes in mice. Similarly, *in vivo* reprogramming of cardiac fibroblasts to induced cardiomyocytes can also be achieved with miRNAs (Jayawardena et al., 2012). The induced cardiomyocytes can attenuate cardiac dysfunction after myocardial infarction. Collectively, these studies demonstrated the use of *in vivo* reprogramming for cardiomyocyte regeneration which would have important therapeutic implications for myocardial infarction.

Another interesting research direction is the use of *in vivo* reprogramming technologies to rejuvenate aging cells. It is widely established that the use of the Yamanaka factors (*Oct4, Sox2, Klf4,* and *c-Myc*) can reprogram somatic cells into iPS cells – a reprogramming process that reverses many aspects of aging signatures in the target cells, including reorganization of mitochondria and reconstituted telomerase activity (Rohani et al., 2014). Overexpression of the Yamanaka factors *in vivo* allowed generation of iPS cells in mice, however this resulted in teratoma formation in multiple organs (Abad et al., 2013). Ocampo et al. (2016) overcame this issue of tumorigenicity using an inducible system to repeatedly induce short-term expression of the Yamanaka factors in mice. Interestingly, this strategy prevented teratoma formation in the mice. Using a premature aging mouse model for Hutchinson-Gilford progeria syndrome, the author showed that this partial reprogramming strategy reversed the cellular and physiological hallmarks of aging, such as DNA damage and epigenetic dysregulation, and strikingly extended the lifespan of these mice by ∼30%. Furthermore, this reprogramming approach also allowed wild type aged mice to increase resistance to metabolic disease and muscle injury. These results supported the use of short-term induction of Yamanaka factors for partial reprogramming to rejuvenate aged cells, and provided exciting possibilities of using regenerative medicine for anti-aging.

## *In Vivo* Reprogramming for Regeneration of the Central Nervous System

*In vivo* reprogramming in the nervous system has advanced rapidly since 2013. Transcription factor-based reprogramming to induce neurogenesis in the brain *in vivo* has tremendous potential as a novel approach for neural repair in an injury or disease setting. For instance, Grande et al. (2013) showed that *Neurog2* can be used for *in vivo* reprogramming to generate new neurons in the neocortex and striatum following a stab wound or ischemia. The addition of FGF2 and EGF was found to enhance this process and generate more neurons *in vivo.* Interestingly, the outcome of this neuronal reprogramming *in vivo* was highly dependent on location; *in vivo* reprogramming in the striatum only induced a small number of neurons, whereas *in vivo* reprogramming in the neocortex induced a large number of immature neurons, but only a small number of neurons matured to a later stage. However, the precise cell types that were targeted for reprogramming remain unclear in this study, as the retroviruses used in this study did not deliver reprogramming factors to a specific cell type in the brain. Future studies with the use of cell type-specific promoter to induce reprogramming will help address this issue.

In the nervous system, many studies have targeted the glial cells for *in vivo* reprogramming into neurons. An early study by Niu et al. (2013) demonstrated *in vivo* neuronal reprogramming with lentiviral delivery of a single transcription factor – *Sox2*. *Sox2* alone was sufficient to reprogram striatal astrocytes into neuroblasts within the adult mouse brain. Subsequent addition of BDNF and noggin can direct the induced neuroblasts to develop into electrophysiologically mature neurons (Niu et al., 2013). Concurrently, Torper et al. (2013) reported a glial-neuron reprogramming approach that did not require treatment with neurotrophic factors. The authors demonstrated that lentiviral delivery of *Ascl1*, *Brn2,* and *Myt1l* into the striatal astrocytes of the mouse brain converted them into neurons, with a reported efficiency of up to ∼6% (Torper et al., 2013). The induced neurons exhibit neuronal morphology and express NeuN, but are otherwise not well-characterized. A follow up study by the same group reported an improved method for neuronal reprogramming (Torper et al., 2015). This improved method utilized AAV delivery of *Ascl1*, *Lmx1a*, and *Nurr1* into striatal NG2 glia, which resulted in induced neurons with functional electrophysiological properties. Using modified rabies virus to trace cell-cell connectivity, the authors demonstrated that the induced neurons successfully integrated into the existing host circuitry, with an average of innervation by 3–4 host neurons. This confirmed synaptogenesis between existing host afferent inputs and the newly formed *in vivo* reprogrammed neurons (Torper et al., 2015). Subsequent study by Pereira et al. (2017) showed that the NG2 glia-derived induced neurons reach functional maturation after 12 weeks following *in vivo* reprogramming, with properties similar to fast-spiking, parvalbumin-containing interneurons.

Whilst the *in vivo* reprogramming studies discussed above targeted quiescent glial cells, other studies targeted the reactive glial cells, which are apparent in disease or injury settings. Using a stab wound injury model to elicit activation of glial cells, Heinrich et al. (2014) showed that sole addition of *Sox2* can induce conversion of reactive glia into induced neurons in the adult mouse cerebral cortex. This demonstrated the potential for using *in vivo* reprogramming for cortical repair of acute injury. Although the induced neurons possess functional electrophysiological properties, the authors noted that many of the induced neurons lack neuronal subtype-specific traits such as neurotransmitter release, which may suggest that the reprogrammed cells are in an immature state. Similarly, Guo et al. (2014) provided experimental evidence that overexpression of *Neurod1* can reprogram reactive glial cells to neurons in the cortex of a stab-injured or Alzheimer’s disease model. This *Neurod1*-mediated reprogramming is highly efficient, with a reported efficiency of > 90%. Interestingly, the authors showed a differential effect of *Neurod1* in the reprogramming of different glial cells. While astrocytes were mostly reprogrammed into glutamatergic neurons, NG2 glial cells were reprogrammed into both glutamatergic and GABAergic neurons. Collectively, these studies demonstrate a potential therapeutic approach to regenerate neurons and restore lost neuronal function in an injured or diseased brain. Moreover, *in vivo* reprogramming strategies targeting reactive glial cells for conversion into neurons may also be used to reduce reactive gliosis and reduce glial scarring following injury in the nervous system (Li and Chen, 2016).

## Using *In Vivo* Reprogramming for Retinal Regeneration

Similar advances have been made for *in vivo* reprogramming in the retina. The major glial cells in the retina, Müller glia (MG), have been shown in a number of species to have the ability to convert into new neurons. In teleost fish and postnatal chickens, upon retinal stresses, MG can dedifferentiate to multipotent progenitors and give rise to all retinal neural cell types to promote retinal regeneration (Fischer and Reh, 2001; Goldman, 2014). In rodents, acute retinal stresses can stimulate MG to proliferate and produce a small number of neurons *in vivo* (Jadhav et al., 2009). These studies support the notion that, given the appropriate stimuli, the MG possess the plasticity to become retinal neurons, and as such are an ideal target for testing the feasibility of *in vivo* reprogramming to regenerate retinal neurons.

Pioneering studies from the Reh laboratory have paved ways for using transcription factor-based reprogramming of MG into retinal neurons *in vivo*. Their work showed that following retinal injury, overexpression of *Ascl1* in transgenic mice can reprogram MG into retinal neurons, including photoreceptors, bipolar and amacrine cells (Ueki et al., 2015). However, this reprogramming is only observed in young mice (day 7) and the neurogenic capacity of MG is lost by day 16. Subsequently, an important improvement was reported by the same laboratory. The addition of a histone deacetylase inhibitor Trichostatin A, combined with *Ascl1* overexpression following retinal injury, allowed *in vivo* reprogramming of MG to neurons in adult mice up to 8 months old (Jorstad et al., 2017). Using an elegant Cre-loxP system, the authors expressed *Ascl1* specifically in MG and traced their conversion to retinal neurons *in vivo*. The authors also provided evidence that addition of Trichostatin A enabled epigenetic changes that allow expression of other retinal neuronal genes, such as *Otx2*, that presumably facilitate this *in vivo* reprogramming process. Notably, the reprogrammed neurons are mostly bipolar cells and are able to form synaptic connections with the existing retinal circuitry and respond to visual stimuli. These results provide a major step toward using *in vivo* reprogramming to regenerate the retina.

A more recent study by Yao et al. (2018) built on this *in vivo* reprogramming strategy to direct MG into rod photoreceptors. Using a two-step reprogramming method, the authors used AAV to first overexpress beta catenin to stimulate MG proliferation, then overexpress a cocktail of *Otx2, Crx,* and *Nrl* for differentiation into rod photoreceptors. The MG-derived rod photoreceptors formed functional synapses and are able to functionally integrate into the visual pathway. Although the reprogramming efficiency is difficult to assess *in vivo*, remarkably the authors showed that this reprogramming process regenerated sufficient rod photoreceptors to restore visual functions in a photoreceptor degeneration mouse model, which included restoration of responses to light and transmission of visual signals to the primary visual cortex. Notably, the demonstration of using AAV for MG reprogramming, as well as functional recovery in a disease model provided a major step toward clinical development of cell therapies to replace retinal neurons, which have important implications in treatment of retinal degenerative diseases.

It is worth noting that the therapeutic potential of *in vivo* reprogramming is not limited to generating cells that were lost in injury or disease. *In vivo* reprogramming can also be used to intervene disease progression. For instance, Montana et al. (2013) demonstrated that transgenic knockout of *Nrl*, an essential transcription factor for the formation of rod photoreceptors during development, can partially reprogram adult mouse rods into cones *in vivo*. Interestingly, by preventing secondary cone loss, this reprogramming strategy rescued cone function and achieved an improvement in the vision of a mouse model of retinitis pigmentosa. Since then, two follow-up studies have confirmed this finding and improved the reprogramming method, by using AAV-mediated CRISPR/Cas9 to knockout *Nrl* and preserve cone functions in multiple mouse models of photoreceptor degeneration (Yu et al., 2017; Zhu et al., 2017). Based on this concept of converting rod into cone photoreceptors, another study has highlighted the potential of developing small molecules to inhibit the rod photoreceptor gene *Nr2e3* to treat retinitis pigmentosa (Nakamura et al., 2016). Collectively, this cone-to-rod reprogramming approach represents an exciting direction with promising results that warrant further investigation.

## Application of Cellular Reprogramming for Neuroregeneration: Clinical Translation and Challenges

In degenerative diseases or injuries that affect the central nervous system, often there is no cure or effective treatment once the neural cells are lost. Regenerative medicine offers a promising approach to replace or regenerate the lost cells to restore normal functions in the patients. Several strategies have been proposed for neuroregeneration (Table 1), including cell replacement therapies using cells from donors, cells derived from pluripotent stem cells, or cells derived from direct reprogramming *in vitro*. Compared to pluripotent stem cell differentiation, direct reprogramming *in vitro* offers a potentially faster and more cost-effective approach to generate patient-specific cells for transplantation. Also, the recent development of *in vivo* reprogramming represents a novel approach for regenerative medicine, by stimulating endogenous cells to replace the cells lost in diseases/injuries. Notably, transcription factor-based reprogramming *in vivo* would follow the clinical development pathway of gene therapy which is highly attractive commercially (de Wilde et al., 2016), as opposed to cell replacement therapy which incurs high cost associated with production of clinical grade cells. It is clear that there are great opportunities of developing novel regenerative strategies using *in vivo* reprogramming. However, there are also significant hurdles that need to be overcome before this technology can translate into the clinics. Here we will discuss some of these hurdles for *in vivo* reprogramming and potential solutions for these issues.

**Table 1:** Comparison of different strategies for neuroregeneration.

Strategy	Category	Preparation/availability	Cost	GMP cell	Advantages	Disadvantages
				facility?
Transplantation using donor cells	Cell based	Dependent on donor availability	$	No	– Allogeneic transplantation – Avoid risks of mutations induced by *in vitro* culture – Suitable for treatment of genetic diseases	– Often donor shortage – Cell quantity limited by donor tissue – Risk of graft rejection


Transplantation using cells derived from donor ESC/iPSC	Cell based	PSC can be differentiated and stored as frozen cells, thawed and expanded on demand	$$	Yes	– Allogeneic transplantation – PSC represent a renewable cellular source – Potential to upscale cell production – A bank of limited number of PSC cell lines can potentially provide HLA matching for a large portion of the population – Suitable for treatment of genetic diseases	– Requires meticulous quality control for derived cells – Risk of teratoma formation – Long time required for iPSC generation and subsequent stem cell differentiation – Risk of mutations arise during *in vitro* culture – Needs HLA matching to address immunorejection issues


Transplantation using cells derived from patient-specific iPSC	Cell based	Requires extraction of patient cells, iPSC generation and subsequent differentiation	$$$$	Yes	– Autologous transplantation – PSC represent a renewable cellular source – Potential to upscale cell production – Potential for development of disease modeling, personalized drug discovery and gene therapy	– Requires meticulous quality control for derived cells – Risk of teratoma formation – Long time required for iPSC generation and subsequent stem cell differentiation – Risk of mutations arise during *in vitro* culture – For genetic diseases would requires combined gene therapy


Transplantation using cells derived from direct reprogramming *in vitro*	Cell based	Requires extraction of patient cells and direct reprogramming *in vitro*	$$	Yes	– Autologous transplantation – Potentially faster than iPSC generation and subsequent differentiation – Less risk of tumorigenicity – Potential for development of disease modeling, personalized drug discovery and gene therapy	– Requires meticulous quality control for derived cells – Risk of mutations arise during *in vitro* culture – Non-renewable cellular source: limited cell quantity – For genetic diseases would requires combined gene therapy


Direct reprogramming *in vivo*	Gene based	Viral vectors can be stored as off-the shelf treatment	$	No	– Bypass transplantation – Less risk of tumorigenicity Avoid risks of mutations induced by *in vitro* culture	– Possible depletion of endogenous cell pool following reprogramming – For genetic diseases would requires combined gene therapy


*PSCs, pluripotent stem cells; ESCs, embryonic stem cells; iPSCs, induced pluripotent stem cells; HLA, human leukocyte antigen.*

### a) Potential Risk of Endogenous Cell Depletion

In order to achieve functional rescue in disease or injury, often a sizeable number of cells must be regenerated. Understandably, this may lead to the depletion of endogenous cells that were reprogrammed. This is an important consideration for using *in vivo* reprogramming for regeneration. To what extent, if any, will normal function be compromised with the repurposing of these cells? Will the remaining population be sufficient to carry out their functional roles? Careful selection of the target cell for reprogramming is therefore necessary. For instance, targeting cell types with a large number of cells, or a cell type whose pool is continually replenished under homeostasis, would be advantageous in the application of *in vivo* reprogramming.

### b) Reprogrammed Cells May Still Harbor Genetic Mutations

In degenerative diseases that are caused by genetic mutation(s), it is important to consider that while *in vivo* reprogramming may regenerate cells that were lost, the reprogrammed cells will still harbor the disease-causing mutation and can be prone to degeneration in the future. In this regard, it can be argued that disease-mediated degeneration may take years to manifest, and as such *in vivo* reprogramming can be used as a temporal treatment. Alternatively, a combination of gene therapy and *in vivo* reprogramming, as demonstrated by Yao et al. (2018) in retinal glial cells, will allow for both the correction of genetic mutation(s) and cell replacement in hereditary degenerative diseases.

### c) Identification of Reprogramming Factors

A major limitation is the identification of reprogramming factors that promote conversion to a specific target cell type. The conventional strategy is to screen through a list of transcription factors that are important for the development of the target cells to identify the factors that allow cellular reprogramming. However, this is a time-consuming and laborious process. To address this issue, several groups have reported on the development of computational algorithm to analyze gene regulatory networks to predict the transcription factors that promote cellular reprogramming. One example is Mogrify, which predicts and ranks transcription factors based on their transcriptional regulatory influence on the target cell type by enrichment and network topology analysis (Rackham et al., 2016). Another recent algorithm incorporates cell cycle features to improve the prediction of reprogramming factors and ideal timing for the introduction of these factors during the direct reprogramming process (Ronquist et al., 2017).

### d) Gene Delivery Systems for Reprogramming Factors

Another consideration for the clinical translation of *in vivo* reprogramming is the gene delivery system. Ideally, delivery of reprogramming factors should be safe, effective, and specific to the target cells. A marker-specific promoter can be incorporated into the vector design to achieve cell specific expression of reprogramming factors and minimize off-target effects of *in vivo* reprogramming. There are numerous methods commonly used to deliver the genes, including lentivirus/retrovirus, adenovirus, AAV, and liposomes. Lentivirus and retrovirus allow delivery of larger insert, but given their nature to integrate into the host genome they are less desirable to be used for gene therapy. Of the integration-free viral delivery systems, AAV is a very attractive gene delivery system for gene therapy, with mounting evidence supporting its safety and effectiveness in preclinical and clinical settings (Naso et al., 2017). Moreover, AAV with different serotypes show varying transduction efficiency that are cell type dependent (Balaji et al., 2013), a characteristic which can be used to promote specific gene expression in a target cell type. However, AAV has a ∼5 kb limit on the insert size (Björklund et al., 2000), thus often this is not suitable for co-expression of more than one transgene. Further development and modification of AAV will be an exciting direction to improve the specificity of transduction in target cells.

Alternatively, another potential solution is to use small molecules to replace transcription factors for *in vivo* reprogramming (Li et al., 2018). Although *in vivo* reprogramming in the neural systems with an all-chemical approach is still in its infancy, this is an attractive reprogramming strategy for clinical translation, as small molecules can be cost-effective, non-immunogenic, produced in high quantity and efficiently delivered across the membrane into cells. Future studies in this direction would be very exciting with high potentials for clinical translation.

### e) Tracking and Analyzing Cellular Reprogramming *in vivo*

It is imperative to distinguish newly reprogrammed cells from the resident cells within the tissue. To address this, the gene delivery vector can be designed to incorporate a fluorescent tag, such as the Cre-loxP system, for lineage tracing of the cell conversion *in vivo*. In many cases, the reprogramming efficiency *in vivo* may be low, thus the analysis techniques utilized have to be sufficiently sensitive to address this. The emergence of single cell analysis provides an exciting technology to understand the process of *in vivo* reprogramming. For instance, the recent advances in microfluidic technology have greatly enhanced the throughput of single cell transcriptome analysis (Stubbington et al., 2017). Single cell transcriptome analysis is highly suitable to analyze the process of cellular reprogramming which is often inefficient. We expect to see more utilization of single cell RNA sequencing in the coming years.

## Concluding Remarks

Rapid advances in the field of cellular reprogramming have created a new paradigm for regenerative medicine. Direct reprogramming provides a feasible method to generate cells *in vitro* for biological studies, tissue engineering or transplantation. Moreover, the application of direct reprogramming *in vivo* provides an exciting regenerative strategy to reprogram and repurpose endogenous cells within damaged tissues, which overcomes limitations associated with transplantation of donor cells. The studies outlined here demonstrate advances in their respective fields and collectively demonstrate an exciting progress of applying cellular reprogramming for regenerative medicine. Future studies to test the therapeutic potential of *in vivo* reprogramming in animal disease models or injury models would be of high interest. We expect that the development of better computational methods would improve prediction of transcription factors to convert cell fates and advance the field of cellular reprogramming. Other emerging new technologies, such as single cell transcriptome analysis and new CRISPR/Cas systems to modulate gene expression, would provide new tools to better understand the cellular reprogramming process, improve the specificity and efficiency of cellular reprogramming and facilitate the clinical application of this technology for regenerative medicine.

## Author Contributions

LF, LEW, and RW provided conceptual framework of this manuscript. LF, LEW, CT, TN, SH, and AH contributed to writing of manuscript.

## Conflict of Interest Statement

The authors declare that the research was conducted in the absence of any commercial or financial relationships that could be construed as a potential conflict of interest.
