# Corrigendum: Potentials of Cellular Reprogramming as a Novel Strategy for Neuroregeneration

**DOI:** 10.3389/fncel.2019.00147

**Published:** 2019-05-03

**Authors:** Lyujie Fang, Layal El Wazan, Christine Tan, Tu Nguyen, Sandy S. C. Hung, Alex W. Hewitt, Raymond C. B. Wong

**Affiliations:** ^1^Centre for Eye Research Australia, East Melbourne, VIC, Australia; ^2^Ophthalmology, Department of Surgery, The University of Melbourne, Melbourne, VIC, Australia; ^3^Department of Ophthalmology, Jinan University, Guangzhou, China; ^4^Menzies Institute for Medical Research, University of Tasmania, Hobart, TAS, Australia; ^5^Shenzhen Eye Hospital, Shenzhen, China

**Keywords:** cell reprogramming, retina, neuroregeneration, direct reprogramming, *in vivo* reprogramming, regenerative medicine, gene therapeutics

In the original article, there was a mistake in Figure 1B as published. The schematic diagram contained an incorrect label of “Pluripotent cells/Neighbouring cells,” the correct label is “Neighbouring cells.” The corrected Figure 1B appears below.

**Figure 1:**
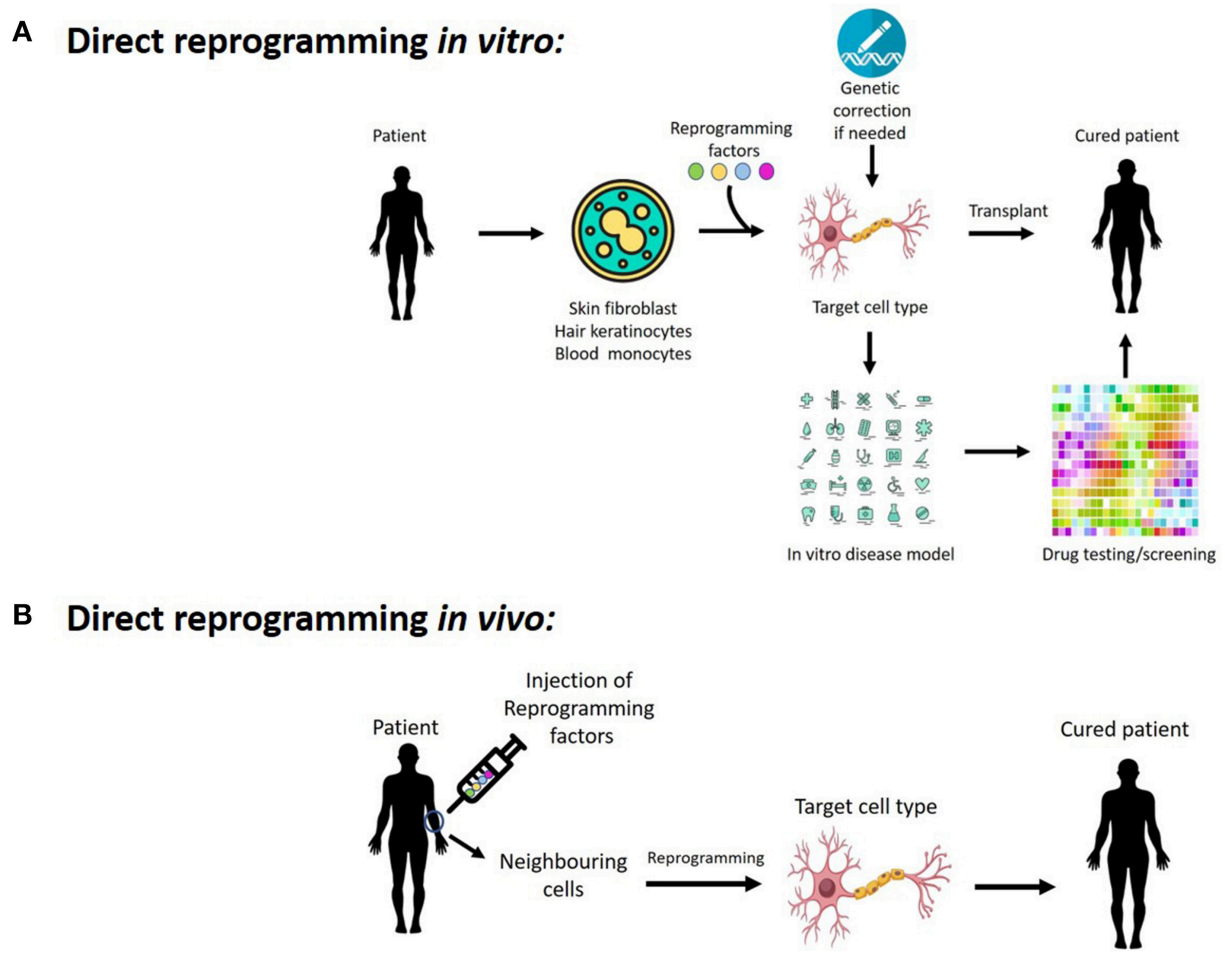
Potentials of cellular reprogramming **(A)** *in vitro* and **(B)** *in vivo* for regenerative medicine, disease modeling, as well as drug discovery and testing gene therapy.

The authors apologize for this error and state that this does not change the scientific conclusions of the article in any way. The original article has been updated.

